# MBX-102/JNJ39659100, a Novel Non-TZD Selective Partial PPAR-*γ* Agonist Lowers Triglyceride Independently of PPAR-*α* Activation

**DOI:** 10.1155/2009/706852

**Published:** 2009-04-23

**Authors:** Apurva Chandalia, Holly J. Clarke, L. Edward Clemens, Bindu Pandey, Vic Vicena, Paul Lee, Brian E. Lavan, Francine M. Gregoire

**Affiliations:** ^1^Department of Biology, Metabolex, Inc., 3876 Bay Center Place, Hayward, CA 94545, USA; ^2^Department of Molecular Biology, Genentech Inc., 1 DNA Way, South San Francisco, CA 94080, USA

## Abstract

MBX-102/JNJ-39659100 (MBX-102) is a selective, partial PPAR-*γ* agonist that lowers glucose in the absence of some of the side effects, such as weight gain and edema, that are observed with the TZDs. Interestingly MBX-102 also displays pronounced triglyceride lowering in preclinical rodent models and in humans. Although in vitro reporter gene studies indicated that MBX-102 acid is a highly selective PPAR-*γ* agonist that lacks PPAR-*α* activity, we sought to determine if PPAR-*α* activation in vivo could possibly contribute to the triglyceride lowering abilities of MBX-102. In vivo studies using ZDF and ZF rats demonstrated that MBX-102 lowered plasma triglycerides. However in ZF rats, MBX-102 had no effect on liver weight or on hepatic expression levels of PPAR-*α* target genes. Further in vitro studies in primary human hepatocytes supported these findings. Finally, the ability of MBX-102 to lower triglycerides was maintained in PPAR-*α* knockout mice, unambiguously establishing that the triglyceride lowering effect of MBX-102 is PPAR-*α* independent. The in vivo lipid lowering abilities of MBX-102 are therefore mediated by an alternate mechanism which is yet to be determined.

## 1. Introduction

The peroxisome proliferator-activated receptors (PPARs) belong to the nuclear hormone receptor superfamily of transcription factors. They are lipid sensors known to govern numerous biological processes. The three PPAR subtypes (*α*, *δ* (*β*), and *γ*) regulate the expression of numerous genes involved in a variety of metabolic pathways [1, 2]. PPAR-*γ* is expressed most abundantly in adipose tissue and is a master regulator of adipogenesis and mediates the anti-diabetic activity of the marketed insulin-sensitizing drugs that belong to the thiazolidinedione (TZD) class-such as rosiglitazone (Avandia) and pioglitazone (Actos). PPAR-*α* is highly expressed in the liver and is the molecular target for the fibrates (e.g., fenofibrate and gemfibrozil), a class of drugs that lower plasma triglycerides and increase HDL levels in humans [3, 4]. The function of PPAR *δ*(*β*) is still not fully understood but recent evidence suggests that this ubiquitously expressed PPAR isoform has pleiotropic actions that may govern diverse physiological processes, including the regulation of lipid and lipoprotein metabolism [5, 6], insulin sensitivity [7], cardiac function [8], epidermal biology [9], neuroprotection [10], and gastrointestinal tract function and disease [11].

As indicated above, the clinical relevance of PPAR-*γ* agonists is highlighted by the currently marketed antidiabetic blockbuster drugs, Avandia, and Actos. These drugs behave as selective PPAR-*γ* full agonists as they are potent and selective activators of PPAR-*γ* [12]. In humans, they enhance insulin action, improve glycemic control with a significant reduction in the level of glycohaemoglobin (HbA_1C _), and have variable effects on serum triglyceride levels in patients with type 2 diabetes [13]. Despite their proven efficacy, they possess a number of deleterious side effects, including significant weight gain and peripheral edema [14–16], increased risks of congestive heart failure, and increased rate of bone fracture [15, 17, 18].

The weight gain associated with the use of TZDs is observed in preclinical species and in humans [15, 19] and is likely due to multiple interacting factors, including increased adiposity and fluid retention [17, 20]. Fluid retention and subsequent edema are the most significant undesired effects of TZD treatment. Edema is a prominent problem in patients taking TZDs particularly those who are also taking insulin or sulfonylureas. In susceptible patients with pre-existing conditions, fluid retention and edema can lead to an increased incidence of congestive heart failure [21]. Moreover the inference that TZD treatment cause a significant increase in the risk of myocardial infarction and an increase in the risk of death from cardiovascular in type 2 diabetic patients was recently made [22, 23], leading the FDA to request the addition of a black box warning to the label of both Actos and Avandia.

Another major side effect of glitazone use is related to their detrimental skeletal actions as they are known to cause bone loss in rodents [24–26]. More importantly, TZDs treatment was recently shown to decrease bone formation and accelerated bone loss in healthy and insulin resistant individuals and/or to increase the fracture rate in diabetic women treated with TZDs [27, 28]. Such major safety concerns have not only restrained the clinical use of these drugs but have also led to development failure of a large number of PPAR agonists [15, 17].

During the last decade, a major investment was made by the pharmaceutical industry to develop safer PPAR agonists (reviewed in [20, 29]). This effort led to the description of several unique TZD-like and non-TZD-like partial PPAR-*γ* agonists that display insulin-sensitizing activity associated with lower stimulation of adipogenesis and therefore with a potential for reduced side effects [15, 17, 20, 30–33].

MBX-102/JNJ39659100 (MBX-102) is a compound in development for the treatment of type 2 diabetes. It is a single enantiomer of halofenate, a drug developed for lipid lowering that was tested clinically in the 1970s as a hypolipidemic and hypouricemic agents [34, 35]. Studies with halofenate in diabetic patients also demonstrated significant effects on plasma glucose and insulin [36, 37], suggesting insulin sensitizing properties. It was recently discovered that both halofenate and MBX-102 are selective partial PPAR-*γ* modulators thereby offering an explanation for their anti-diabetic properties and lack of weight gain and edema [20, 38].

The results presented here show, in agreement with the published halofenate data, that MBX-102 also displays significant triglyceride lowering in preclinical rodent models. As triglycerides lowering in preclinical species and in humans is often considered a hallmark of PPAR-*α* activation and because the mechanism of action by which halofenate lowers triglycerides has not been elucidated, we performed a series of studies to assess if PPAR-*α* activation could possibly play a role in the hypolipidemic efficacy of MBX-102.

## 2. Material and Methods

### 2.1. Chemicals

MBX-102, pioglitazone, and rosiglitazone maleate were synthesized at Metabolex (Metabolex Inc, Hayward, CA). Fenofibrate and GW7647 were obtained from Sigma-Aldrich (Saint-Louis, MO). WY-14643 was obtained from Eagle Picher Pharmaceutical Services (Lenexa, KS).

### 2.2. Cell-Based Reporter Assays

The determination of mouse PPAR-*α*, *δ*, and *γ* activation was performed as previously described [38]. Briefly, HEK-293T cells were transfected with Gal4 chimeras and reporter gene plasmids using Lipofectamine 2000 (InVitrogen, Carlsbad, CA) and incubated for 4 hours before treatment with compound for 20–24 hours. Expression was assayed using the Steady-Glo assay system (Promega, Madison, WI).

### 2.3. Human Primary Hepatocytes

Cryo-preserved primary human hepatocytes were obtained from Celsis (Baltimore, MD). Cells were quickly thawed in a 37°C water bath and placed into 5 mL of warm InvitroGRO CP medium (Celsis Baltimore, MD) with 2.2% Torpedo antibiotic (Celsis Baltimore, MD). A total of 350 000 cells/well were plated in 24-well collagen-coated plates (Becton Dickinson, San Jose, CA) and incubated overnight. The following day the media was replaced with fresh InvitroGRO HI medium (Celsis Baltimore, MD) containing either DMSO (0.5%) or the test compounds, and the cells were incubated for 24 hours. Cells were then harvested and processed for gene expression analysis. Total RNA was isolated using Trizol (Invitrogen, Carlsbad, CA), and cDNA was prepared by reverse transcription using the High Capacity cDNA Reverse Transcription Kit (Applied Biosystems, Foster City, CA). RT-PCR (Taqman) was performed in 96-well plates containing Taqman fast universal PCR master mix (Applied Biosystems, Foster City, CA) and the appropriate gene expression assay mixes for human HADHB, HMGCS2, CYP4a11, and RPLP0 (Applied Biosystems, Foster City, CA). The “fold change versus vehicle” in gene expression was calculated using the comparative *C*_*t*_ method for relative quantification. For each compound, two to five independent experiments were performed, and in each experiment the compounds were tested in at least 2 replicate wells. The “fold change versus vehicle” data for replicate experiments were pooled prior to statistical analysis.

### 2.4. In Vivo Studies

The Metabolex Institutional Animal Care and Use Committee approved all animal care and experimental procedures described below. All animals were housed in temperature (22 ± 3°C) and humidity (55 ± 4%) controlled rooms, with 12 hour light (6AM-6PM)/dark cycle. Unless specified otherwise, mice were housed 4 to 5 mice/cage, and rats were housed 2 rats/cage and were allowed ad libitum access to tap water and Purina Rodent Chow (Laboratory Rodent Diet 5001, St. Louis, Mo., USA).

#### 2.4.1. Reagents and Assays

Plasma glucose levels were measured using the method of Trinder [39] (Glucose Oxidase G7016, Peroxidase P8125, Sigma Chemical Co., St. Louis, MO). Plasma triglycerides were measured using a triglyceride Diagnostic Kit (Sigma Chemical Co., MO). Plasma-free fatty acid (FFA) levels were measured using the HR Series NEFA-HR [2] (Wako, Richmond, VA). Plasma insulin levels were determined using either a rat or a mouse insulin EIA kit (ALPCO Chem. Windham, NH).

#### 2.4.2. Zucker Diabetic Fatty Rat Study

9 week-old Zucker diabetic fatty (ZDF) rats were obtained from Charles River (Boston, MA). Vehicle and drug suspensions were administered to the rats daily by oral gavage for 11 days. Six rats were assigned to each of the following groups: Vehicle (10 mL/kg), rosiglitazone maleate (4 mg/kg), and MBX-102 (100 mg/kg). Body weight and food intake were recorded weekly. On day 11, rats were fasted for 6 hours and blood samples (~500 *μ*L) were collected via cardiac puncture at the time of necropsy.

#### 2.4.3. Zucker Fatty Rat Study

10 week-old male Zucker Fatty (ZF) rats were obtained from Harlan (Indianapolis, IN). Vehicle and drug suspensions were administered to the rats daily by oral gavage for 32 days. Eight rats were assigned to each of the following groups: ZF Vehicle (5 mL/kg), ZF + fenofibrate (450 mg/kg), and ZF + MBX-102 (100 mg/kg). Body weight and food intake were recorded every 2 or 3 days in the fed state until day 28 of the study. At day 33 (24 to 28 hours post-last dose), blood samples were collected following a 6 hour fast from each rat via cardiac puncture for total triglyceride and insulin determinations. Liver weights were also recorded. Following necropsy, a small (~100–200 mg) section of liver was excised, placed into a cryovial and immediately frozen in liquid nitrogen. Tissue homogenates for gene expression analysis were prepared as follows: frozen liver samples were placed into a 2 mL homogenization vial containing HTG tissue lysis buffer (1 mL/100 mg of tissue, High Throughput Genomics, Tucson, AZ) and a 5 mm steel bead. Tissues were homogenized for 5 minutes (25 pulses/second) in a Qiagen Tissue Lyser. Homogenates were heated at 95°C for 10 minutes, frozen at −80°C, and shipped to high throughput genomics (HTG, Inc., Tuscson, AZ) for mRNA measurement using a custom qNPA multiplex array. The HTG quantitative nuclease protection (qNPA) technology was used to analyze changes in mRNA expression levels. All raw values were obtained by imaging with a high-resolution imager and were normalized against two endogenous house keeping genes, RPL10a (rat ribosomal protein L10A) and Arbp (rat acidic ribosomal phosphoprotein P0). For the treatment groups, the fold changes (FC) were calculated using the Vehicle-treated values as 100% (FC = 1).

#### 2.4.4. PPAR-*α* KO Study

Male wild-type (C57BL/6N) and PPAR-*α* knockout mice (B6.129S4-*Ppara* ^*tm*1*Gonz*^, on C57BL/6N background, N12) were received from Taconic (Germantown, New-York) at 4–6 weeks of age. Animals were allowed access ad libitum to tap water and Rodent Chow (RD D12450B, New Brunswick, NJ). Ten wild-type (WT) and 10 knockout (KO) mice were assigned to each of the following groups: vehicle (5 mL/kg), WY-14643 (130 mg/kg), and MBX-102 (200 mg/kg). Compounds or vehicle were delivered by oral gavage once daily for 7 days. At the end of the drug treatment, blood samples from each mouse were collected, following a 6 hour fast, via cardiac puncture for total triglyceride and free fatty acid determinations. Three independent studies were performed to evaluate the ability of MBX-102 to lower triglycerides in WT and KO mice. Datasets obtained from the 3 studies were pooled prior to statistical analysis.

### 2.5. Statistical Analysis

Data are expressed as mean ± SEM. Prism software (GraphPad v 5.01, San Diego, CA) was used for all statistical analyses. Unless specified otherwise in the figure legends, 1-way ANOVA followed by either Tukey's multiple comparison test or Newman-Keul multiple comparison test or 2-way ANOVA followed by Bonferroni post test was used to assess statistical differences between groups. All *P*-values of less than .05 were considered statistically significant.

## 3. Result

**Figure 1:**
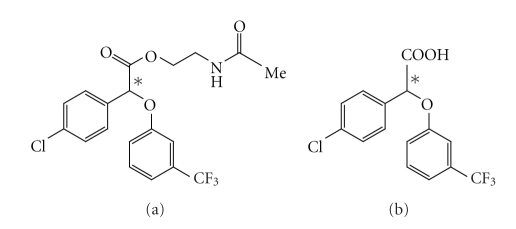
Chemical structures of the prodrug ester (a) and active-free acid form (b).

MBX-102/JNJ-39659100 (Figure 1(a)) is the (–) enantiomer of halofenate, a drug previously described as a partial PPAR-*γ* agonist [38]. MBX-102 is a prodrug ester (Figure 1(a)), that is rapidly and completely modified in vivo by non-specific serum esterases to the mature free acid form MBX-102 acid (Figure 1(b)), which is the circulating form of the drug. For these reasons MBX-102 was utilized for in vivo studies, whereas the acid form was utilized for all in vitro studies.

**Figure 2:**
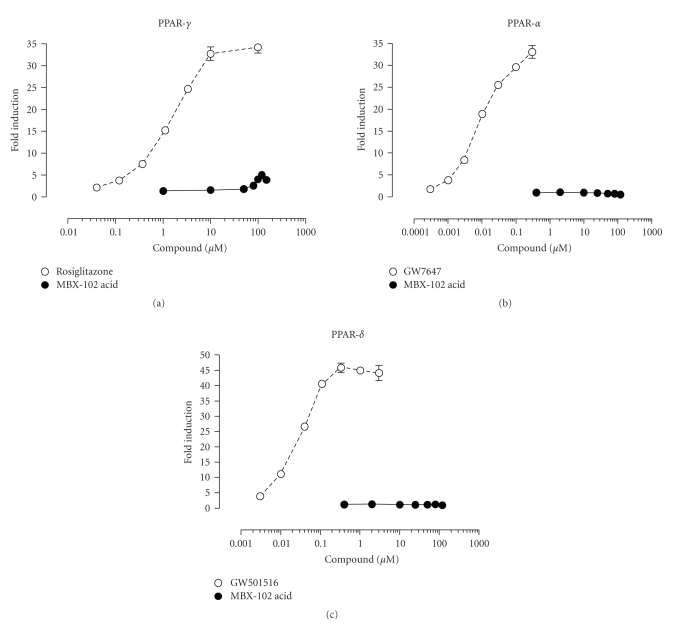
Gal4 Reporter assay data for mouse PPAR-*γ* (a), mouse PPAR-*α* (b), and mouse PPAR-*δ* (c). Values are plotted as mean ± SEM and are representative of at least 2 independent experiments.

As previously described for halofenate, cell-based in vitro studies revealed that MBX-102 acid also behaves as a selective, weak partial PPAR-*γ* agonist. As shown in Figure 2(a), a dose-dependent activation of mouse GAL4-PPAR-*γ* was observed in response to MBX-102 acid and rosiglitazone, with EC_50_s of ~12 *μ*M for MBX-102 acid and ~1.5 *μ*M for rosiglitazone. Compared to the full agonist rosiglitazone, MBX-102 acid was a much weaker transactivator of PPAR-*γ*, as indicated by its lower transactivation activity (~10% of that observed with rosiglitazone). MBX-102 acid selectivity toward PPAR-*γ* was confirmed by the lack of transactivation of mouse GAL4-PPAR-*α* or *δ* (Figures 2(b) and 2(c)). A similar PPAR activation profile of MBX-102 acid was also observed for human and rat PPARs, including selectivity for PPAR-*γ*, partial agonism, and similar EC_50_s for PPAR-*γ* activation (data not shown).

**Figure 3:**
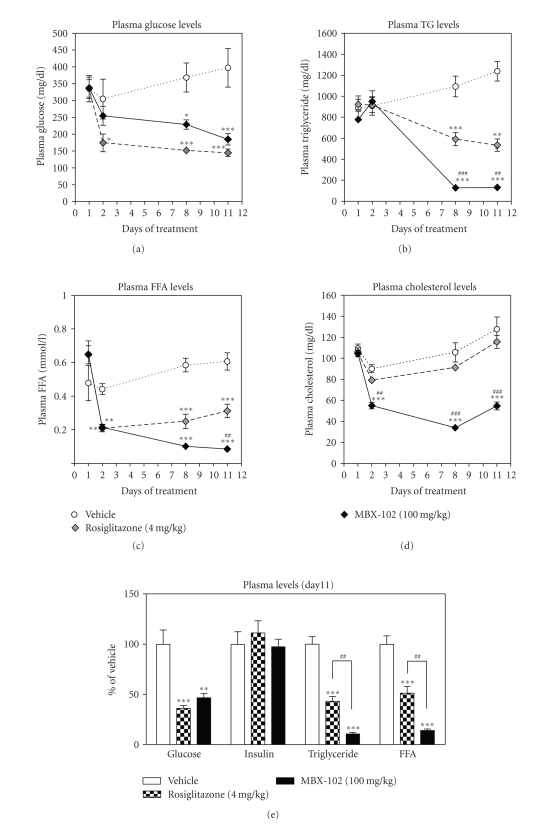
Effect of MBX-102 (100 mg/kg) and rosiglitazone (4 mg/kg) on fasting plasma glucose (a), triglycerides (b), FFA (c), and cholesterol (d) levels during the course of treatment of male ZDF rats. Values are plotted as mean ± SEM (∗: *P* < .05, ∗∗: *P* < .01, ∗∗∗: *P* < .001 versus ZDF vehicle; *#*: *P* < .05, *##*: *P* < .01, *###*: *P* < .001 versus MBX-102-treated group, 2-way ANOVA followed by Bonferroni post tests). (e) Fasting plasma glucose, insulin, triglycerides, and FFA levels on day 11. Values are plotted as mean percentage of vehicle ± SEM (NS: *P* > .05, ∗: *P* < .05, ∗∗: *P* < .01, ∗∗∗: *P* < .001 versus ZDF vehicle, *##*: *P* < .01 versus MBX-102-treated group, 1-way ANOVA and Tukey's multiple comparison test).

**Figure 4:**
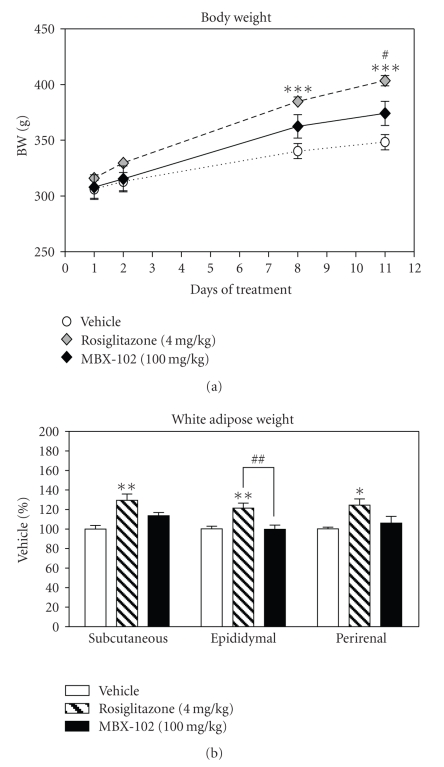
Effect of MBX-102 (100 mg/kg) and rosiglitazone (4 mg/kg) on body weight (BW) (a) and white adipose tissue weights (b) after 11 days of treatment of male ZDF rats. For the adipose tissue weight, the values are plotted as mean percentage of vehicle ± SEM (∗: *P* < .05, ∗∗: *P* < .01, ∗∗∗: *P* < .001 versus ZDF Vehicle; *#*: *P* < .05, *##*: *P* < .01 versus MBX-102, (a) 2-way ANOVA and Bonferroni post tests or (b) 1-way ANOVA and Tukey's multiple comparison test).

Halofenate was initially developed as a hypolipidemic agent, and MBX-102 is reported to share this ability. In order to assess MBX-102 efficacy we evaluated the lipid lowering properties of MBX-102 as well as its antidiabetic effects, using the male Zucker Diabetic Fatty (ZDF) rat model. ZDF rats were treated with MBX-102 (100 mg/kg) or rosiglitazone (4 mg/kg) for 11 days. As shown in Figure 3, after a 6 hours fast, MBX-102 significantly decreased triglyceride (Figure 3(b)), free fatty acid (Figure 3(c)), and cholesterol (Figure 3(d)) levels. The magnitude of reduction in these lipid parameters was significantly higher than what was observed for rosiglitazone (TG 89% versus 57%; FFA 86% versus 49% and Cholesterol 57% versus 10%, for MBX-102 and rosiglitazone, resp.), suggesting superior hypolipidemic activity of MBX-102 compared to rosiglitazone. Moreover, both MBX-102 and rosiglitazone significantly reduced fasting blood glucose (Figures 3(a) and 3(e)), confirming that MBX-102 is an efficacious antidiabetic agent. This effect was anticipated as antidiabetic properties including glucose lowering, and insulin sensitization in preclinical models is a hallmark of full PPAR-*γ* agonists and has also been reported for partial agonists [20]. In addition, significant increases in body weight (Figure 4(a)) and adipose tissue weight (Figure 4(b)) were observed with rosiglitazone treatment only, indicating that MBX-102 does not display the classical weight gain effects of the full PPAR-*γ* agonists.

**Figure 5:**
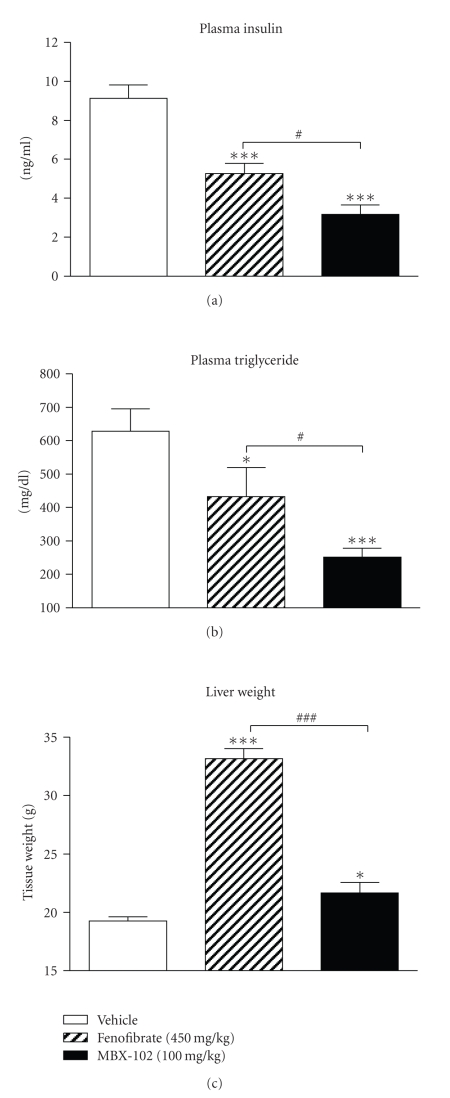
Effect of MBX-102 (100 mg/kg) and fenofibrate (450 mg/kg) on fasting plasma insulin (a), triglycerides (b), and liver weights (c) after 32 days of treatment of male ZF rats. Values are plotted as mean ± SEM (∗: *P* < .05, ∗∗∗: *P* < .001 versus ZF vehicle, *#*: *P* < .05, *###*: *P* < .001, MBX-102 versus fenofibrate, 1-way ANOVA, and Newman-Keuls multiple comparison test).

In order to evaluate further the lipid lowering ability of MBX-102, male Zucker Fatty (ZF) rats, a well-established model for hypertriglyceridemia and obesity, were used. The PPAR-*α* agonist fenofibrate, a known triglyceride lowering agent, was included in the study as a comparator. As ZF rats are hyperinsulinemic and insulin resistant, the insulin sensitizing effect of MBX-102 was also assessed. ZF male rats were treated with either vehicle, fenofibrate (450 mg/kg) or MBX-102 (100 mg/kg) for 32 days. In this study, no significant differences in body weight or food intake were observed upon drug treatment (data not shown). As shown in Figure 5(a), both MBX-102 and fenofibrate treatment significantly lowered fasting plasma insulin after 32 days of treatment. However, the reduction observed for MBX-102-treated ZF rats was significantly greater when compared to the reduction observed for the fenofibrate-treated animals. In this rat model, MBX-102 robustly decreased fasting plasma triglycerides after 32 days of treatment (Figure 5(b)). Although fenofibrate also led to a reduction in plasma triglyceride levels, the reduction was less pronounced when compared to MBX-102 (31% versus 60%, Figure 5(b)).

**Figure 6:**
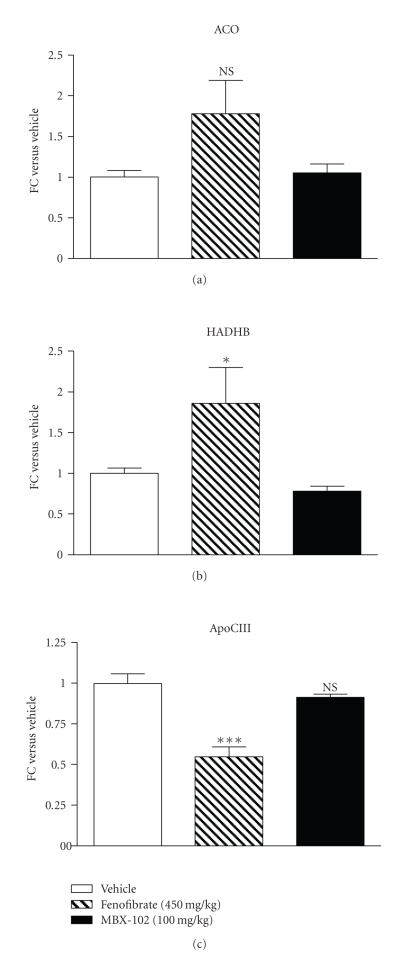
Gene expression levels of PPAR-*α* responsive genes in livers derived from male ZF rats treated for 32 days with either MBX-102 (100 mg/kg) or fenofibrate (450 mg/kg). Expression levels of ACO (a), HADHB (b), and apoC-III (c) mRNA. Values represent mean ± SEM (NS: *P* > .05, ∗∗∗: *P* < .001 versus Vehicle-treated, 1-way ANOVA, and Newman-Keuls multiple comparison test).

To determine if PPAR-*α* activation might be responsible for the triglyceride lowering ability of MBX-102, liver weight and liver gene expression levels of several PPAR-*α* responsive genes were assessed in this study. As shown in Figure 5(c), fenofibrate treatment markedly increased liver weight while MBX-102 treatment caused minimal change in this parameter. In addition, a slight but not statistically significant upregulation of ACO (Figure 6(a)), significant upregulation of HADHB (Figure 6(b)), and significant downregulation of apoC-III (Figure 6(c)) mRNA levels were also detected upon treatment with fenofibrate. In contrast, MBX-102 treatment had no effect on the mRNA expression levels of these three PPAR-*α* responsive genes, suggesting that MBX-102 lowered triglycerides independently of PPAR-*α* activation.

**Figure 7:**
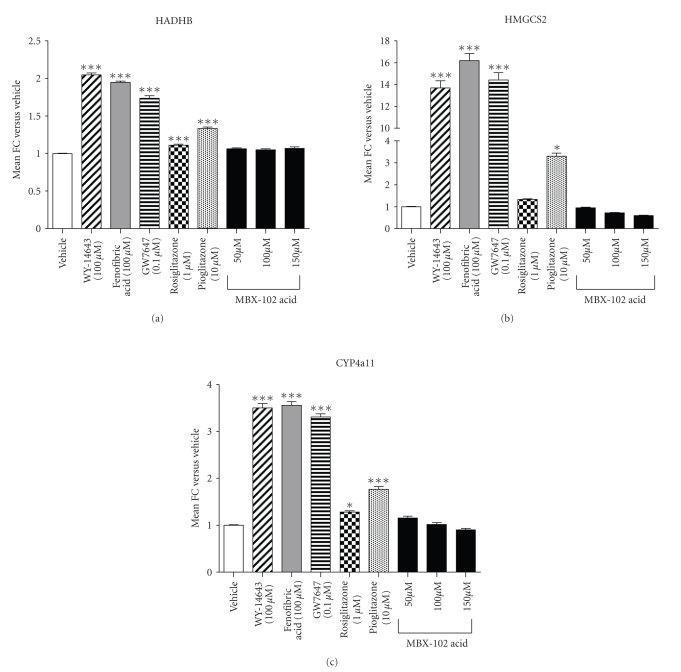
Effect of PPAR-*α* agonists, rosiglitazone, pioglitazone, and MBX-102 acid on HADHB (a), HMGCS2 (b), and CYP4a11 (c) mRNA levels in primary human hepatocytes. Values represent mean ± SEM (∗: *P* < .05, ∗∗∗: *P* < .001 versus Vehicle-treated, 1-way ANOVA and Tukey's multiple comparison test).

In order to further explore the PPAR selectivity of MBX-102 in a physiologically relevant cell-based system, primary human hepatocytes were used to evaluate the expression levels of several PPAR-*α* responsive genes. Primary human hepatocytes were treated with known PPAR-*α* agonists including GW7647, WY-14643, and fenofibric acid as well as with the PPAR-*γ* agonists rosiglitazone, pioglitazone, and MBX-102 acid. As shown in Figure 7, HADHB (a), HMGCS2 (b), and CYP4a11 (c) mRNA levels were significantly upregulated by treatment with all PPAR-*α* agonists. The extent of upregulation was similar for all three PPAR-*α* agonists. Interestingly, these three genes were also significantly up-regulated by pioglitazone although the magnitude of this effect was less than for the three PPAR-*α* agonists. In contrast, although MBX-102 acid treatment was able to induce mRNA levels of the PPAR-*γ* responsive genes CD36 and FABP4 in these cells (data not shown), it had no effect on any of the PPAR-*α* responsive gene tested supporting the in vivo results observed in the ZF rats.

**Figure 8:**
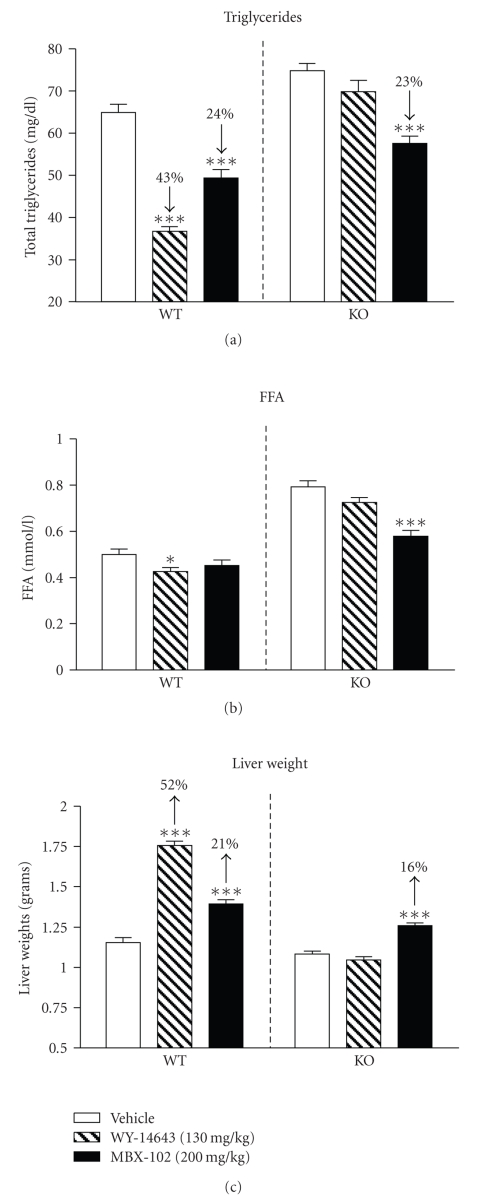
Effect of MBX-102 (200 mg/kg) and WY-14643 (130 mg/kg) on plasma triglyceride levels (a), FFA levels (b), and liver weights (c) in WT and KO mice after 7 days treatment of PPAR-*α* KO mice. Values represent mean ± SEM (∗: *P* < .05, ∗∗∗: *P* < .001 versus Vehicle, 2-way ANOVA, and Bonferroni post-tests).

Based on these results, we speculated that MBX-102 would be able to lower triglycerides in mice lacking PPAR-*α*. Therefore, the effect of MBX-102 on triglyceride levels was evaluated in wild-type (WT) and PPAR-*α* knockout (KO) mice. WT and KO mice were treated with either vehicle, the PPAR-*α* selective agonist WY-14643 (130 mg/kg), or MBX-102 (200 mg/kg) for 7 days. Prior to evaluating triglyceride lowering, single, and repeated doses, pharmacokinetic analyses were performed with both compounds in both WT and KO mice, and no difference in plasma drug exposure was observed (data not shown). As shown in Figure 8(a), treatment with WY-14643 significantly reduced plasma triglycerides in WT mice. This effect was totally abolished in the PPAR-*α* KO mice, confirming that PPAR-*α* was required for this effect. In contrast, a significant reduction in plasma triglycerides was observed upon treatment with MBX-102 both in WT and PPAR-*α* KO mice, demonstrating this effect was independent of PPAR-*α* activation. Plasma FFA levels in WT and KO mice are depicted in Figure 8(b). Compared to vehicle-treated WT mice, plasma FFA levels were markedly elevated in vehicle-treated KO mice. Treatment with WY-14643 had little (WT) to no effect (KO) on plasma FFA levels. In contrast, although MBX-102 had no impact on FFA levels in WT mice, it led to significant FFA lowering in the KO animals (Figure 8(b)). At the end of the study changes in liver weight upon compound treatment were evaluated. As expected, treatment with WY-14643 increased liver weight by 52% in WT mice, and the effect was totally abolished in the PPAR-*α* KO mice (Figure 8(c)). MBX-102 treatment mildly increased liver weight to a similar extent in both WT and KO mice, indicating this effect occurred independently of PPAR-*α* activation.

## 4. Discussion

Type 2 diabetes mellitus is a chronic disease characterized by glucose intolerance, hyperinsulinemia, and dyslipidemia, [40]. PPAR-*γ* agonists such as rosiglitazone and pioglitazone belong to the thiazolidinedione (TZD) class and are currently in clinical use for lowering glucose levels in diabetes [41, 42].

Our results show that MBX-102 acid, a non-TZD PPAR agonist, is a partial, selective PPAR-*γ* agonist which has the potential to offer antidiabetic efficacy comparable to rosiglitazone. More importantly, compared to rosiglitazone, treatment of ZDF rats with MBX-102 did not significantly affect body weight and white adipose tissue mass, suggesting that in humans, MBX-102 will not display the classical adverse effects of the full PPAR-*γ* agonists [15, 20]. These data are in agreement with a previously published report that established that halofenate, the racemic mixture from which MBX-102 is derived, had comparable insulin sensitization to rosiglitazone in the absence of body weight gain [38].

Among the efficacy parameters measured in our studies, the most differentiating feature of MBX-102 was its impressive lipid lowering abilities. MBX-102 was much more efficacious than rosiglitazone and fenofibrate at lowering plasma triglycerides in the diabetic, insulin-resistant rat models tested. In rodents, differences in feeding behavior can induce significant fluctuation in plasma triglycerides and free fatty acid levels. Such an artifact can be excluded in the present studies as all measurements were performed on 6 hour post-fasting plasma samples.

In the clinical setting, fibrate therapy is known to achieve significant triglyceride lowering, an expected feature of PPAR-*α* agonists [43, 44]. In contrast, the lipid effects of the marketed PPAR-*γ* agonists are not as clear, as pioglitazone displays beneficial effects on lipid profile in diabetic patients while rosiglitazone does not [13, 45]. Our data suggest that MBX-102 will display beneficial effects on lipid profile in humans, and this was recently confirmed in a phase 2a clinical trial [46]. Overall, these results are not unexpected based on the history of halofenate, the parent molecule from which MBX-102 was derived. Halofenate was tested clinically in the 1970s as a hypolipidemicand hypouricemic agent and was shown to lower serum triglycerides and uric acid in patients with a variety of hyperlipidemias [36, 37, 47–49].

Although the mechanism by which halofenate and MBX-102 reduce triglycerides in preclinical rodent models and in humans remains unclear, a major concern was that MBX-102 may exert its hypolipidemic action through PPAR-*α* activation. As mentioned above, triglyceride lowering is a *well-known* feature of PPAR-*α* agonists. Although the classical in vitro reporter gene assays we used to assess MBX-102 selectivity toward PPAR-*γ* clearly show their inability to transactivate human, mouse, or rat PPAR-*α*, the biological relevance of these assays remains unclear as they do not truly represent the interaction between the ligand and its receptor in a physiologically relevant setting [17]. The discontinuation of several dual *α*/*γ* PPAR agonists at mid to late stage of development due to major safety concerns including dose-limiting toxicities and carcinogenicity-related issues clearly highlights the potential for increased risk of safety liabilities for dual agonists compared to selective agonists [15, 17]. The carcinogenic risk is of particular interest as duals agonists appear to have enhanced rodent carcinogenicity potential compared to selective gamma agonists (http://www.fda.gov/cder/present/DIA2004/Elhage.ppt), increasing the burden of developing such agents for use in humans. Therefore in order to demonstrate that MBX-102 can lower triglycerides independently of PPAR-*α* activation, we undertook a series of studies in which we used physiologically relevant readouts of PPAR-*α* activation.

Although both fenofibrate and MBX-102 had the ability to modulate triglyceride levels in ZF rats, MBX-102 only had a small effect on rat liver, which is unlikely mediated by PPAR-*α* activation as MBX-102 treatment led to a similar liver weight increase in PPAR-*α* KO mice. Moreover, MBX-102 was unable to regulate the hepatic expression levels of the 3 known PPAR-*α* target genes tested, suggesting its inability to transactivate rat PPAR-*α* in vivo. In contrast, the anticipated regulation of these genes (i.e., upregulation of 2 key genes involved in fatty acid oxidation and downregulation of apoC-III) was observed with fenofibrate [50, 51].

Primary human hepatocytes represent a biologically relevant cell line to model clinical effects of PPAR-*α* agonism and therefore were used to further explore the PPAR selectivity of MBX-102 acid. In this cell-based system, we were unable to detect any induction of PPAR-*α* responsive genes upon MBX-102 acid treatment, further confirming its lack of PPAR-*α* activity. Moreover, the finding that MBX-102 still lowers triglycerides in PPAR-*α* deficient mice unambiguously demonstrates that MBX-102 can lower triglycerides effectively in the absence of PPAR-*α*.

Although these results corroborate that MBX-102 is a selective PPAR-*γ* agonist, the mechanism by which it lowers triglycerides in preclinical species and in the clinic still needs to be addressed. Pioglitazone also possesses triglyceride lowering effects in humans but in this case partial contribution of PPAR-*α* activation cannot be ruled out. Our hepatocyte data indeed show that pioglitazone upregulates PPAR-*α* responsive genes, in agreement with published reports showing that pioglitazone binds to and activates the human PPAR-*α* receptor [52]. Moreover, pioglitazone has recently been shown to raise hepatic apoA-I and HDL through a PPAR-*α*-dependent pathway [53].

Studies performed in the 1970s with halofenate may provide a potential clue as to how MBX-102 lowers triglycerides. In normal rats, sustained reduction of serum triglyceride levels upon treatment with halofenate was suggested to be mediated through the inhibition of hepatic triglyceride formation. Although the mechanism of action mediating this effect is not yet elucidated, it was also suggested that the inhibition of hepatic triglyceride formation might be related to drug-induced decreases in the availability of fatty acids for triglyceride synthesis [54]. Our results in ZDF rats are in agreement with this hypothesis as a marked lowering of circulating free fatty acids was indeed observed upon MBX-102 treatment.

Taken as a whole, the data from these studies provide definitive evidence that MBX-102 acid does not activate PPAR-*α*. As such, the lowering of triglycerides in vivo by MBX-102 is not a PPAR-*α* mediated effect, but is rather mediated by an alternate mechanism which has yet to be determined. Additional studies are required to determine if MBX-102, like halofenate, is capable of inhibiting liver triglyceride formation. More importantly, studies designed to understand how such inhibition may occur will be required. Among these, measurement of serum and hepatic triglyceride formation and turnover will be necessary.

## References

[1] Feige JN, Gelman L, Michalik L, Desvergne B, Wahli W (2006). From molecular action to physiological outputs: peroxisome proliferator-activated receptors are nuclear receptors at the crossroads of key cellular functions. *Progress in Lipid Research*.

[2] Michalik L, Auwerx J, Berger JP (2006). International Union of Pharmacology. LXI. Peroxisome proliferator-activated receptors. *Pharmacological Reviews*.

[3] Kersten S (2008). Peroxisome proliferator activated receptors and lipoprotein metabolism. *PPAR Research*.

[4] Staels B, Dallongeville J, Auwerx J, Schoonjans K, Leitersdorf E, Fruchart J-C (1998). Mechanism of action of fibrates on lipid and lipoprotein metabolism. *Circulation*.

[5] Sprecher DL (2007). Lipids, lipoproteins, and peroxisome proliferator activated receptor-*δ*. *The American Journal of Cardiology*.

[6] Sprecher DL, Massien C, Pearce G (2007). Triglyceride: high-density lipoprotein cholesterol effects in healthy subjects administered a peroxisome proliferator activated receptor *δ* agonist. *Arteriosclerosis, Thrombosis, and Vascular Biology*.

[7] Reilly SM, Lee C-H (2008). PPAR*δ* as a therapeutic target in metabolic disease. *FEBS Letters*.

[8] Madrazo JA, Kelly DP (2008). The PPAR trio: regulators of myocardial energy metabolism in health and disease. *Journal of Molecular and Cellular Cardiology*.

[9] Schmuth M, Jiang YJ, Elias PM, Feingold KR (2008). Thematic review series: skin lipids. Peroxisome proliferator-activated receptors and liver X receptors in epidermal biology. *Journal of Lipid Research*.

[10] Iwashita A, Muramatsu Y, Yamazaki T (2007). Neuroprotective efficacy of the peroxisome proliferator-activated receptor *δ*-selective agonists in vitro and in vivo. *Journal of Pharmacology and Experimental Therapeutics*.

[11] Peters JM, Hollingshead HE, Gonzalez FJ (2008). Role of peroxisome-proliferator-activated receptor *β*/*δ* (PPAR*β*/*δ*) in gastrointestinal tract function and disease. *Clinical Science*.

[12] Willson TM, Cobb JE, Cowan DJ (1996). The structure-activity relationship between peroxisome proliferator-activated receptor *γ* agonism and the antihyperglycemic activity of thiazolidinediones. *Journal of Medicinal Chemistry*.

[13] Deeg MA, Tan MH (2008). Pioglitazone versus rosiglitazone: effects on lipids, lipoproteins, and apolipoproteins in head-to-head randomized
clinical studies. *PPAR Research*.

[14] Bavirti S, Ghanaat F, Tayek JA (2003). Peroxisome proliferator-activated receptor-gamma agonist increases both low-density lipoprotein cholesterol particle size and small high-density lipoprotein cholesterol in patients with type 2 diabetes independent of diabetic control. *Endocrine Practice*.

[15] Rubenstrunk A, Hanf R, Hum DW, Fruchart J-C, Staels B (2007). Safety issues and prospects for future generations of PPAR modulators. *Biochimica et Biophysica Acta*.

[16] Yang T, Soodvilai S (2008). Renal and vascular mechanisms of thiazolidinedione-induced fluid retention. *PPAR Research*.

[17] Shearer BG, Billin AN (2007). The next generation of PPAR drugs: do we have the tools to find them?. *Biochimica et Biophysica Acta*.

[18] Grey A (2008). Skeletal consequences of thiazolidinedione therapy. *Osteoporosis International*.

[19] Fonseca V (2003). Effect of thiazolidinediones on body weight in patients with diabetes mellitus. *The American Journal of Medicine*.

[20] Zhang F, Lavan BE, Gregoire FM (2007). Selective modulators of PPAR-*γ* activity: molecular aspects related to obesity and side-effects. *PPAR Research*.

[21] Nesto RW, Bell D, Bonow RO (2004). Thiazolidinedione use, fluid retention, and congestive heart
failure: a consensus statement from the American Heart Association and American Diabetes Association. *Diabetes Care*.

[22] Lincoff AM, Wolski K, Nicholls SJ, Nissen SE (2007). Pioglitazone and risk of cardiovascular events in patients with type 2 diabetes mellitus: a meta-analysis of randomized trials. *The Journal of the American Medical Association*.

[23] Nissen SE, Wolski K (2007). Effect of rosiglitazone on the risk of myocardial infarction and death from cardiovascular causes. *The New England Journal of Medicine*.

[24] Lazarenko OP, Rzonca SO, Hogue WR, Swain FL, Suva LJ, Lecka-Czernik B (2007). Rosiglitazone induces decreases in bone mass and strength that are reminiscent of aged bone. *Endocrinology*.

[25] Rzonca SO, Suva LJ, Gaddy D, Montague DC, Lecka-Czernik B (2004). Bone is a target for the antidiabetic compound rosiglitazone. *Endocrinology*.

[26] Sottile V, Seuwen K, Kneissel M (2004). Enhanced marrow adipogenesis and bone resorption in estrogen-deprived rats treated with the PPARgamma agonist BRL49653 (rosiglitazone). *Calcified Tissue International*.

[27] Meier C, Kraenzlin ME, Bodmer M, Jick SS, Jick H, Meier CR (2008). Use of thiazolidinediones and fracture risk. *Archives of Internal Medicine*.

[28] Schwartz AV (2008). TZDs and bone: a review of the recent clinical evidence. *PPAR Research*.

[29] Balint BL, Nagy L (2006). Selective modulators of PPAR activity as new therapeutic tools in metabolic diseases. *Endocrine, Metabolic and Immune Disorders Drug Targets*.

[30] Carmona MC, Louche K, Lefebvre B (2007). S 26948: a new specific peroxisome proliferator-activated receptor *γ* modulator with potent antidiabetes and antiatherogenic effects. *Diabetes*.

[31] Einstein M, Akiyama TE, Castriota GA (2008). The differential interactions of peroxisome proliferator-activated receptor *γ* ligands with Tyr473 is a physical basis for their unique biological activities. *Molecular Pharmacology*.

[32] Gelman L, Feige JN, Desvergne B (2007). Molecular basis of selective PPAR*γ* modulation for the treatment of type 2 diabetes. *Biochimica et Biophysica Acta*.

[33] Chang F, Jaber LA, Berlie HD, O'Connell MB (2007). Evolution of peroxisome proliferator-activated receptor agonists. *Annals of Pharmacotherapy*.

[34] Aronow WS, Harding PR, Khursheed M, Vangrow JS, Papageorge's NP (1973). Effect of halofenate on serum uric acid. *Clinical Pharmacology and Therapeutics*.

[35] Aronow WS, Harding PR, Khursheed M, Vangrow JS, Papageorge's NP, Mays J (1973). Effect of halofenate on serum lipids. *Clinical Pharmacology and Therapeutics*.

[36] Feldman EB, Gluck FB, Carter AC (1978). Effects of halofenate on glucose tolerance in patients with hyperlipoproteinemia. *Journal of Clinical Pharmacology*.

[37] Krut LH, Seftel HC, Joffe BI (1977). Comparison of clofibrate with halofenate in diabetics with hyperlipidaemia. *South African Medical Journal*.

[38] Allen T, Zhang F, Moodie SA (2006). Halofenate is a selective peroxisome proliferator-activated receptor*γ* modulator with antidiabetic activity. *Diabetes*.

[39] Trinder P (1969). Determination of blood glucose using an oxidase-peroxidase system with a non-carcinogenic chromogen. *Journal of Clinical Pathology*.

[40] Stumvoll M, Goldstein BJ, van Haeften TW (2005). Type 2 diabetes: principles of pathogenesis and therapy. *The Lancet*.

[41] Campbell IW (2005). The clinical significance of PPAR gamma agonism. *Current Molecular Medicine*.

[42] Waugh J, Keating GM, Plosker GL, Easthope S, Robinson DM (2006). Pioglitazone: a review of its use in type 2 diabetes mellitus. *Drugs*.

[43] Remick J, Weintraub H, Setton R, Offenbacher J, Fisher E, Schwartzbard A (2008). Fibrate therapy: an update. *Cardiology in Review*.

[44] Duriez P (2003). Mechanisms of actions of statins and fibrates. *Therapie*.

[45] Goldberg RB, Kendall DM, Deeg MA (2005). A comparison of lipid and glycemic effects of pioglitazone and rosiglitazone in patients with type 2 diabetes and dyslipidemia. *Diabetes Care*.

[46] Rosenstock J, Flores-Lozano F, Schartz S, Gonzalez-Galvez G, Karpf D (2005). MBX-102: a novel non-TZD insulin sensitizer that improves glycemic control without causing edema or weight gain in patients with type 2 diabetes (T2DM) on concomitant insulin therapy. *Diabetes*.

[47] Aronow WS, Vicario MD, Moorthy K, King J, Vawter M, Papageorge's NP (1975). Long term efficacy of halofenate on serum triglyceride levels. *Current Therapeutic Research, Clinical and Experimental*.

[48] Dujovne CA, Azarnoff DL, Huffman DH, Pentikäinen P, Hurwitz A, Shoeman DW (1976). One year trials with halofenate, clofibrate, and placebo. *Clinical Pharmacology and Therapeutics*.

[49] Dujovne CA, Azarnoff DL, Pentikainen P, Manion C, Hurwitz A, Hassanein K (1976). A two year crossover therapeutic trial with halofenate and clofibrate. *The American Journal of the Medical Sciences*.

[50] Patsouris D, Reddy JK, Müller M, Kersten S (2006). Peroxisome proliferator-activated receptor *α* mediates the effects of high-fat diet on hepatic gene expression. *Endocrinology*.

[51] Staels B, Vu-Dac N, Kosykh VA (1995). Fibrates downregulate apolipoprotein C-III expression independent of induction of peroxisomal acyl coenzyme A oxidase. A potential mechanism for the hypolipidemic action of fibrates. *The Journal of Clinical Investigation*.

[52] Sakamoto J, Kimura H, Moriyama S (2000). Activation of human peroxisome proliferator-activated receptor (PPAR) subtypes by pioglitazone. *Biochemical and Biophysical Research Communications*.

[53] Qin S, Liu T, Kamanna VS, Kashyap ML (2007). Pioglitazone stimulates apolipoprotein A-I production without affecting HDL removal in HepG2 cells: involvement of PPAR-*α*. *Arteriosclerosis, Thrombosis, and Vascular Biology*.

[54] Cenedella RJ, Crouthamel WG (1976). Halofenate and clofibrate: mechanism of hypotriglyceridemic action in the rat. *Journal of Lipid Research*.

